# Simultaneous functional imaging using fPET and fMRI

**DOI:** 10.1186/2197-7364-2-S1-A66

**Published:** 2015-05-18

**Authors:** Marjorie Villien

**Affiliations:** CERMEP, France

Brain mapping of task-associated changes in metabolism with PET has been accomplished by subtracting scans acquired during two distinct static states. We have demonstrated that PET can provide truly dynamic information on cerebral energy metabolism using constant infusion of FDG and multiple stimuli in a single experiment. We demonstrate here that the functional PET (fPET-FDG) method accomplished simultaneously with fMRI, can enable the first direct comparisons in time, space and magnitude of hemodynamics and oxygen and glucose consumption. The imaging studies were performed on a 3T Tim-Trio MR scanner modified to support an MR-compatible BrainPET insert. Ten healthy subjects were included. The total PET acquisition and infusion time was 90 minutes. We did 3 blocks of right hand fingers tapping for 10 minutes at 30, 50 and 70 minutes after the beginning of the PET acquisition. ASL and BOLD imaging were acquired simultaneously during the motor paradigm. Changes in glucose utilization are easily observed as changes in the TAC slope of the PET data (FDG utilization rate) and in the derivative signal during motor stimuli in the activated voxels. PET and MRI (ASL, and BOLD) activations are largely colocalized but with very different statistical significance and temporal dynamic, especially in the ipsilateral side of the stimuli. This study demonstrated that motor activation can be measured dynamically during a single FDG PET scan. The complementary nature of fPET-FDG to fMRI capitalizes on the emerging technology of hybrid MR-PET scanners. fPET-FDG, combined with quantitative fMRI methods, allow us to simultaneously measure dynamic changes in glucose utilization and hemodynamic, addressing vital questions about neurovascular coupling.

